# Current and Future Perspectives of PD-1/PDL-1 Blockade in Cancer Immunotherapy

**DOI:** 10.1155/2021/6661406

**Published:** 2021-02-22

**Authors:** Rangarirai Makuku, Neda Khalili, Sepideh Razi, Mahsa Keshavarz-Fathi, Nima Rezaei

**Affiliations:** ^1^Cancer Immunology Project (CIP), Universal Scientific Education and Research Network (USERN), Tehran, Iran; ^2^School of Medicine, Tehran University of Medical Sciences, Tehran, Iran; ^3^Department of Immunology, School of Medicine, Tehran University of Medical Sciences, Tehran, Iran; ^4^Student Research Committee, School of Medicine, Iran University of Medical Sciences, Tehran, Iran; ^5^Research Center for Immunodeficiencies, Children's Medical Center, Tehran University of Medical Sciences, Tehran, Iran; ^6^Cancer Immunology Project (CIP), Universal Scientific Education and Research Network (USERN), Sheffield, UK

## Abstract

Cancer immunotherapy, which reactivates weakened immune cells of cancer patients, has yielded great success in recent years. Among immunotherapeutic agents, immune checkpoint inhibitors have been of particular interest and have gained approval by the FDA for treatment of cancers. Immune checkpoint blockade through targeting programmed cell death protein-1 (PD-1) has demonstrated promising antitumor effects in cancer immunotherapy of many different solid and hematologic malignancies. However, despite promising results, a favorable response is observed only in a fraction of patients, and there is still lack of a single therapy modality with curative ability. In this paper, we review the current and future perspectives of PD-1/L1 blockade in cancer immunotherapy, with a particular focus on predictive biomarkers of response to therapy. We also discuss the adverse events associated with PD-1/L1/2 inhibitors, ranging from severe life-threatening conditions such as autoimmune myocarditis to mild and moderate reactions such as skin rashes, and explore the potential strategies for improving the efficacy of immunotherapy with PD-1/L1 checkpoint inhibitors.

## 1. Introduction

Currently, cancer is the second leading cause of death globally, after cardiovascular diseases, claiming one in every six deaths [1]. Immunotherapy, an approach of using drugs that potentiate the natural body's immune system to fight neoplasms, has contributed significantly to cancer treatment in recent years [2]. Among different types of immunotherapy, immune checkpoint inhibitors that target cytotoxic T-lymphocyte antigen-4 (CTLA-4) and programmed cell death protein-1 (PD-1) are of particular interest and play a crucial role in the future of immunotherapy [3]. PD-1/PD ligand (PDL)-1 blocking antibodies function as tumor-suppressing agents via modulation of the interaction between immune cells and tumor cells [4]. Notable clinical efficacy, long-lasting response, and low toxicity of PD-1 checkpoint blockade have been observed in various malignancies [4, 5]. However, dependence on PD-1/L1 monotherapy has proven to be a shortcoming in various aspects, including immune-related adverse events, tumor resistance, tumor relapse, and high costs. In this paper, we will review the current and future perspectives of PD-1/L1 blockade in cancer immunotherapy, and discuss the potential strategies for improving the efficacy of immunotherapy with PD-1/L1 checkpoint inhibitors.

## 2. Overview of the History of PD-1 Immunotherapy

The history of PD-1 discovery dates back to 1992, when Ishida et al. [6] first described the then-novel member of the immunoglobulin gene superfamily, PD-1, on murine immune cells, and reported that PD-1 can induce a classical type of programmed cell death [6]. A few years later, in 1999, researchers demonstrated that PD-1-knockout mice developed lupus-like autoimmune syndromes, signifying that PD-1 acts as an immune checkpoint [7, 8]. In 2014, the Food and Drug Administration (FDA) approved the first anti-PD-1 monoclonal antibody, nivolumab, for second-line treatment of unresectable or metastatic melanoma [9]. Since then, several other anti-PD-1/L1 antibodies have been approved by the FDA for cancer therapy [10]. Most recently, on June 10, 2020, the FDA approved nivolumab (OPDIVO, Bristol-Myers Squibb Co.) for patients with unresectable advanced, recurrent, or metastatic esophageal squamous cell carcinoma (ESCC) after prior fluoropyrimidine- and platinum-based chemotherapy [11].

## 3. Mechanisms of Action of Immune Checkpoint Inhibitors

**Figure 1:**
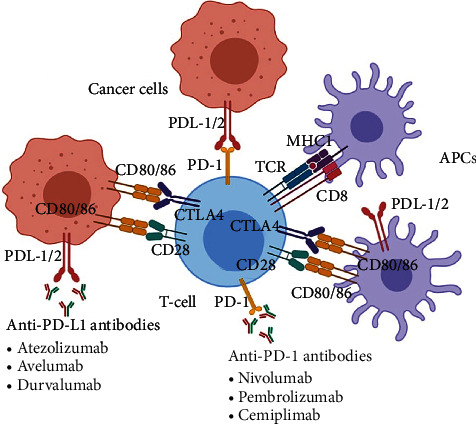
Mechanism of action of PD-1/L1 checkpoint blockade. PD-1: programmed cell death protein-1; TCR: T cell receptor; PDL-1/2: programmed death-ligand 1/2; APC: antigen presenting cell; MHC1: major histocompatibility complex 1; CTLA4: cytotoxic T-lymphocyte-associated protein 4.

Immune checkpoints such as PD-1 are normal control pathways in immune cells, which monitor over activity of the immune system [9, 12, 13]. PD-1 is a member of the CD28 family and an inhibitory receptor expressed on activated T cells, B cells, macrophages, regulatory T cells (Tregs), and natural killer (NK) cells. This receptor has two binding ligands, PDL-1 and PDL-2 (B7 family) that are expressed on T cells, B cells, macrophages, dendritic cells, and many other cells [9, 14]. Binding of PD-1 to either one of the ligands inhibits T cell activity, induces T cell tolerance, suppresses proliferation, reduces the immune response of T cells, and induces cell death [15–17] (Figure 1).

Tumors cause overstimulation of the PD-1/L1 signaling pathway to reduce T cell activation and antigen-specific T cell immune response, thereby bypassing immune surveillance [9, 18, 19]. In addition to expressing PDL-1/2 [20, 21], cancer cells also activate intrinsic cellular signals that enhance cancer cell survival, regulate stress responses, and build tumor resistance against proapoptotic stimuli such as interferons [21–23]. PD-1/L1/2 checkpoint inhibitors block this pathway [24], increasing immune cell proliferation, and enhancing the efficacy of the body's natural antitumor surveillance system [20, 25, 26].

## 4. The Regulatory Effects of PD-1/L1 Inhibition on T Cell Function

### 4.1. Inhibition of T cell Receptor (TCR) Signaling

**Figure 2:**
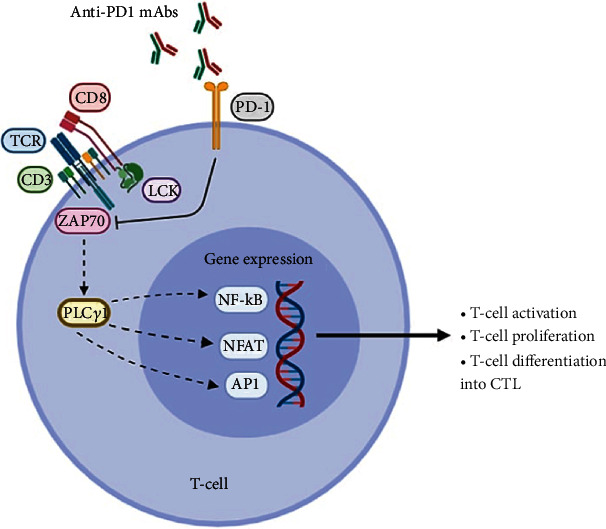
The regulatory effects of PD-1 inhibition on downstream signaling pathways. TCR: T cell receptor; PD-1: programmed cell death protein-1; ZAP70: zeta-chain-associated protein kinase 70; AP1: activator protein 1; NF-*κ*B: nuclear factor kappa-light-chain-enhancer of activated B cells; NFAT: nuclear factor of activated T cells; PLC*γ*: phosphoinositide phospholipase C-*γ*; LCK: lymphocyte-specific protein-tyrosine kinase; mAbs: monoclonal antibodies; CTL: cytotoxic T-lymphocyte.

The PD-1 receptor interacts with its ligands, PDL-1 and PDL-2, to cause an inhibitory effect on the costimulatory function of CD80-CD28, resulting in the downregulation of TCR signal transduction and CD28 costimulation even at very low PD1 expression levels [27]. The PD-1 immunoreceptor tyrosine-based switch motif (ITSM) attaches to Src homology region 2-containing protein tyrosine phosphatase 2 (SHP-2), close to TCR. This interaction blocks the activation of TCR proximal kinases, causing a decrease in Lck-mediated phosphorylation of the TCR CD3*ζ* chains and zeta-chain-associated protein kinase *70 (*ZAP-70), and the initiation of downstream events [28, 29]. This process eventually inhibits cytokine production, T cell proliferation, and survival through downregulating transcription of the prosurvival factor Bcl-X_L_ [27, 30] (Figure 2).

### 4.2. Suppression of the Ras/MEK/ERK Pathway

The Ras/mitogen-activated and extracellular signal-regulated kinase (MEK)/extracellular signal-regulated kinase (ERK) signaling pathway is capable of promoting proliferation and malignant transformation of cells by activating cell growth factors and preventing apoptosis [31]. This pathway is activated by calcium and diacylglycerol, followed by downstream activation of RasGRP1, which then stimulates PLC-*γ*1 [23, 27]. PD-1 suppresses this pathway by inhibiting the activation of PLC-*γ* and Ras [23, 27], leading to suppression of T cell proliferation and subsequent apoptosis.

### 4.3. Inhibition of the PI3K/Akt Signaling Pathway

The PI3K/Akt pathway is important in cell proliferation, cell cycle regulation, apoptosis, and many other pathophysiological processes, which perform a key role in the development of tumors [32]. The inhibitory effect of PD-1 on PI3K/Akt signaling pathway in T cells is recognized as primarily prominent in oncogenesis by stimulating downstream targets of antiapoptosis, cell propagation, and metastasis [27, 33]. PD-1/L1 checkpoint inhibitor therapy invigorates exhausted CD8+ T cells in gastrointestinal stromal tumors via blocking the PI3K/Akt/mTOR signaling pathway [34]. Inhibition of the PI3K/Akt pathway through PD-1 involves phosphatase and tensin homolog (PTEN) phosphorylation and phosphatase activity, facilitated by CK2 Ser380/Thr382/Thr383 cluster. Furthermore, dephosphorylation of the lipid signaling intermediate PIP_3_ by *PTEN*, a tumor suppressor gene, results in repression of Akt functionality, a pivotal process in the PI3K axis [35]. Recent studies have revealed that the PI3K/Akt signaling axis is an effective inhibitor of Treg development [36].

In summary, PD-1 activates signals that suppress specific signaling pathways in T cells, leading to T cell anergy or exhaustion, and thus inhibits T cell immune response [37]. The basis of using immune checkpoint inhibitors such as PD-1 or PD-L1 blocking antibodies in cancer therapy is to enhance T cell-mediated antitumor immune responses and to generate functional tumor-specific CTLs capable of killing tumor cells [37, 38].

## 5. PD-1/L1 Blockade in Patients with Various Malignancies

Since the arrival of PD-1/PDL-1 blockade drugs on the market in 2014, their greatest clinical benefit has been observed among patients with melanoma [9, 14, 39]. These drugs are now considered as first-line treatment for patients with melanoma and have proven to be better than traditional drugs such as dacarbazine in terms of increasing overall survival [9, 14] (Table 1).

In the management of advanced metastatic melanoma, pembrolizumab has improved the overall survival of patients, with relatively minimal toxicity [44].

Apart from melanoma, PD-1 inhibitors have also shown clinical efficacy in both solid and liquid cancers such as bladder, pancreatic, and non-small-cell lung cancer (NSCLC), follicular B cell, and non-Hodgkin lymphoma [45] (Table 2). Both nivolumab and pembrolizumab have provided encouraging results with respect to increased survival benefit and safety in NSCLC patients compared with other drugs such as docetaxel [46]. Evidence suggests that highly immune-cell infiltrate tumors show a better prognosis compared with desert tumors [47]. Tumors with high mutation capacity and antigenicity including tumors with high microsatellite instability (MSI), high tumor mutation load (TML), and mismatch repair deficiencies (dMMR) are also good candidates for PD-1 blockade treatment regimens [48–53]. However, cancers that have a favorable response to PD-1/L1/2 inhibitors are not completely distinguishable by biomarkers [22].

## 6. Clinical Superiority of PD-1/L1 Blockade over Conventional Cancer Therapies

Compared with other cancer therapies, the advantage of immunotherapy lies in the functionality of the immune system; the adaptability and specificity of established immunotherapy to a specific tumor makes a lasting memory of similar antigenic provocation [45]. Additionally, monotherapies of PD-1 inhibitors are associated with higher overall survival rates than monotherapies of other immune checkpoint inhibitors such as ipilimumab (anti-CTLA-4 monoclonal antibody) and BRAF/MEK inhibitors; also, they can be used for the treatment of a variety of cancers [54, 55]. Moreover, the PD-1 blocker, pembrolizumab, is now recognized as a new standard of care and frontline drug for the treatment of ipilimumab-refractory melanoma [56, 57]. There is substantive scientific evidence from various studies indicating that the toxicity associated with PD-1 blocking agents is less than the toxicity associated with other immunotherapies such as interleukin-2 and CTLA-4 blockade [14, 58]. Generally, immunotherapy including PD-1/L1 blockade is safer when compared with other oncotherapy approaches, including irradiation, chemotherapy, and surgery, since it is noninvasive and natural, as the treatment mechanism lies at capacitating self immune cells to fight against neoplasia [48, 54, 59]. Checkpoint immunotherapy is highly specific to the target cells [60]; hence, accompanied by less hazardous side effects [61]. Furthermore, immunotherapy can keep cancer antigen memory [62, 63].

## 7. Disadvantages of PD-1/L1 Inhibitors in Cancer Management

### 7.1. PD-1/L1-Induced Immune-Related Adverse Events

PD-1/L1-induced immune-related adverse events (irAEs) are one of the drawbacks of this type of oncotherapy. Unlike anti-CTLA-4 agents, anti-PD-1/L1 antibodies produced fatal xenogeneic hypersensitivity reactions in a murine model of breast cancer after repeated PD-1/L1 antibody administration [64]. Moreover, anti-PD-1 antibodies have the potential of causing a myriad of immune-related side effects in various organs and systems, including the pancreas, skin, liver, gastrointestinal tract, endocrine, and renal system [65]. Generally, the toxicity associated with PD-1/L1 agents is reported to be less than that with anti-CTLA-4 antibodies, although certain organ-specific side effects, such as pneumonitis, have only appeared under PD-1 blockade therapy [66, 67]. PD-1 blockade-associated pneumonitis is an important adverse event, which is mainly seen in patients with NSCLC [68–71]. In addition, systemic effects, such as meningoradiculitis, polyradiculitis, cardiac arrhythmia, asystole, and paresis, have been noted with PD-1/L1 treatment [72]. Furthermore, severe side effects such as acute heart failure, resulting from myocarditis and induced by the PD-1 inhibitor pembrolizumab, have been reported in patients [73].

### 7.2. Disease Recurrence and Development of Progressive Disease (Hyperprogression)

PD-1 blocker agents are reported to be associated with higher rates of disease recurrence compared with CTLA-4 inhibitors [12]. Although most patients treated with checkpoint inhibitors eventually develop progressive disease [74, 75], up to 18% of patients treated with checkpoint inhibitors can develop oligoprogression; however, local therapy can offer long-lasting progression-free survival (PFS) in some of these patients [75]. Identifying predictors of disease recurrence or hyperprogression is of significant importance in order not to treat patients who might not benefit from receiving immune checkpoint inhibitors [76, 77]. Clinicians are encouraged to consider balancing the risks of toxicity with potential benefits and to make such decisions based on data from challenging patient populations, such as patients with autoimmune disorders, organ transplant, chronic viral infections, ongoing immunosuppressant use, organ dysfunction, pregnancy, brain metastases and impaired functional status [78].

### 7.3. Nonspecific Biomarkers

The expression of PDL-1 is currently a validated and important predictive biomarker; however, this alone is not sufficient and specific enough for determining which patients should be offered PD-1/L1 blockade therapy [59, 79, 80]. In addition, PDL-1 single-nucleotide polymorphisms (SNPs), such as rs4143815 and rs2282055, have been used to further select the final responders [81]. The hypothesis that a tumor expressing the highest level of PDL-1/2 is the most responsive to therapy has not been approved yet [82]. Similarly, MSI and dMMR have been suggested as predictive biomarkers of response to anti-PD-1/L1 antibodies, irrespective of tumor type [83]; however, these phenotypes are also not sufficient to fully predict drug response since they are frequently observed in many cancers and therefore lack specificity [49, 84]. Such findings request for more research to identify novel predictive biomarkers of response to therapy.

The predictive value of PDL-1 expression alone as a biomarker is currently insufficient to understand the depth of tumor immune landscape and deserves further investigation to identify other markers such as TIL, TMB, the genetic and epigenetic variation of IFN-*γ*, circulating biomarkers, and gut microbiota [22]. Based on the current evidence, tumors exhibiting a high PDL-1 expression level as well as TMI, MSI, or dMMR have higher response rates to PD-1 blockade [85]. Also, the quantity as well as the quality of tumor-infiltrating lymphocytes (TILs) is an indicator of response to checkpoint inhibitor therapy [86].

Several studies reported that copy number gains (CNGs) in chromosome 9p24 involving PD-L1 were identified in many cancer types, including lung cancer, melanoma, bladder cancer, head and neck cancer, cervical cancer, soft tissue sarcoma, prostate cancer, gastric cancer, ovarian cancer, and triple-negative breast cancer [87, 88]. In Hodgkin lymphoma, Reed–Sternberg cells and Epstein Bar virus infection stimulate the PD-L1 CNG leading to overexpression of PD-L1 receptors [89]. This shows that PD-L1 CNG is a potential predictive biomarker of response to PD-1 blockade, as witnessed in patients with relapsed or refractory Hodgkin lymphoma, primary mediastinal large B cell lymphoma, and diffuse large B cell lymphoma [89].

Another potential approach to predicting and monitoring clinical response to immunotherapy involves enzymatic deglycosylation of the natural heavily glycosylated PD-1 molecules on cell surfaces during immunohistochemistry. This method, also known as sample deglycosylation, improves anti-PD-L1 antibody binding affinity and signal intensity for easy detection, leading to more precise PD-L1 quantification and prediction of the clinical outcome on bioassays [90].

### 7.4. Cost-Effectiveness of PD-1 Blockade

From the economic perspective, PD-1 blockade monotherapies are generally more expensive than other immunotherapy regimens and conventional cancer therapies [55]. Globally, advanced age is a significant risk factor for developing cancer, which could be attributed to the general decline in immune cell generation and atrophy of the major immunogenic organs with an increase in age. This interprets the large number of elderly patients with cancer [91], highlighting a burden placed on older individuals whose income may be relatively lower than the rest of the population. Kelly and Davar stated that the two problems of checkpoint inhibitors are physical toxicity (irAEs) and financial toxicity [92]. Introducing more specific biomarkers will improve the cost-effectiveness of treatment through optimal patient selection and may escalate the usage of checkpoint blockade [93]. In addition, there is still debate regarding the cost-effectiveness of combination therapy over monotherapy with immune checkpoint inhibitors.

### 7.5. PD-1/L1 Blockade Therapy in Patients with an Underlying Primary Immunodeficiency and/or Autoimmunity

When using immunotherapy, one critical group of patients are those who already have defects in their primary immunity [94]. Defects of the immune system, involving both hyperactivity and hypoactivity, or even complete malfunctioning, all put patients at risk [94, 95]. Various immunotherapies have been used to treat these immune deficiencies [96, 97], but applying cancer immunotherapy to patients with underlying primary immune deficiencies, such as antibody deficiency disorders and hereditary angioedema, has been problematic [94]. In addition, due to the irAEs associated with immune checkpoint inhibitor agents, patients with autoimmune diseases are generally not considered as potential candidates for receiving these types of therapies. Recent studies showed that patients with autoimmune diseases may benefit from immune checkpoint inhibitor therapy; however, the occurrence of irAEs was associated with shorter survival rates [98].

B cell auto-reactivity is more common among patients with certain underlying conditions, such as thyroid abnormalities (thyroiditis and hypothyroidism) and diabetes. Abdel-Wahab et al. found that patients with preexisting autoimmune disease suffer more disease flares when treated with anti-PD-1/PD-L1 agents compared with *de novo* irAEs reported with anti-CTLA-4 agents [99]. Evidence of higher toxicity with CTLA-4 inhibitors than with PD-1/PD-L1 inhibitors is attributed to their “global” activation of naïve and memory T cells from the lymph nodes, unlike PD-1/PD-L1 inhibitors, which modulate T cell activity “locally” in the peripheral tissues [100].

### 7.6. Resistance to Immune Checkpoint Blockade

Although PD-1 signaling blockade significantly enhances antitumor response, produces a long-lasting clinical response, and prolongs survival in some cases, approximately 30%–60% of patients show no response to PD-1/PD-L1 blockade [101]. So far, several mechanisms of resistance to immune checkpoint blockade have been investigated, such as defects in class I antigen presentation, the Wnt/*β*-catenin pathway, and defects in interferon signaling [102]. In addition, emerging adaptive resistance against PD-1/PDL-1 receptor blockers has been reported [4, 23, 103]. In a study by Stenehjem et al., resistance to anti-PD-1 treatment and T cell immunoglobulin and mucin domain-containing molecule-3 (TIM-3) overexpression occurred once the PD-1/PDL-1 pathway was blocked [104].

## 8. Potential Strategies for Improving the Efficacy of PD-1/L1 Blockade Therapy

### 8.1. Identifying Novel Specific Biomarkers for Prediction of Response to Therapy

We recommend prospective studies to focus on the predictive biomarkers of response to immunotherapy, especially PD-1 blockade, which can anticipate the relative treatment efficacy before drug administration. This will minimize the required cost and time, as well as reducing the adverse events related to these drugs that may result in worse disease prognosis [105].

The use of PDL-1 as a biomarker needs more combinations to make it more specific for selecting which patients fit best for single PD-1 therapy or combined therapy. The combination of PDL-1 and T cell infiltration assessment or interferon- (IFN-) gamma gene signature could be a promising suggestion for future studies on predictive biomarkers of PD-1/L1 therapy [106]. Since highly mutated tumors show a favorable response to therapy, more insight is needed at the gene level to determine the mutations which are proresponsive and thus have the potential to be used as predictive biomarkers [107].

Several other potential biomarkers have been recently investigated in the context of response to immunotherapies targeting the PD-1/L1 axis that include the following:

#### 8.1.1. The Gut Microbiome

Recent studies have enriched the understanding of the role played by the gut microbiota in cancer progression and response to immunotherapy. These studies have shown that the gut microbiome may determine the clinical efficacy of PD-1-based immunotherapies in cancer patients. A murine study by Routy et al. demonstrated that antibiotics against the commensal *Akkermansia muciniphila* inhibited the efficacy of anti-PD-1 immunotherapy, while oral supplementation with the same bacterium after fecal microbiota transplantation (FMT) from nonresponding patients improved the clinical outcomes in mouse models of renal cell carcinoma (RCC) and NSCLC [108]. They also observed that antibiotic use in patients with RCC and NSCLC reduced the quantity of *Akkermansia muciniphila*, which potentiates PD-1 blockade by stimulating IL-12 secretion from dendritic cells and promoting the recruitment of CCR9+ CXCR3+ CD4+ T lymphocytes into mouse tumor beds [108]. Similar findings were observed in the gut microbiome profile of patients with hepatocellular carcinoma (HCC) [109] and melanoma [110] who were treated with anti-PD-1 immunotherapy. These results present an essential opportunity for the gut microbiome to be used as a potent predictor and also modulator of response to anti-PD-1 immunotherapy in cancer patients. Currently, studies are investigating the clinical application of manipulating the gut microbiota by using methods such as prebiotics, probiotics, FMT, or capsule loaded with bacteria with the intention of enhancing clinical response in cancer patients treated with PD-1-based immunotherapies. In addition, the mechanisms by which the commensal microbiome modulates response to checkpoint blockade immunotherapy need to be further elucidated [111].

#### 8.1.2. Peripheral Blood Biomarkers

Several studies highlighted that elevated pretreatment neutrophil to lymphocyte ratio (NLR) values were associated with shorter overall survival in patients with metastatic melanoma treated with either ipilimumab or nivolumab [112, 113]. NLR was also found to be correlated with survival in patients with NSCLC and RCC receiving immune checkpoint blockade [114]. Other peripheral blood biomarkers, such as absolute lymphocyte count (ALC), absolute neutrophil count (ANC), and absolute eosinophil count (AEC), were related to survival in melanoma patients undergoing treatment with immune checkpoint inhibitor therapy [115, 116]. Such findings suggest that these routinely collected host-related biomarkers could be used as predictors of response to PD-1-based immunotherapy prior to initiation of treatment [117].

#### 8.1.3. Circulating MicroRNAs

Growing evidence shows that epigenetic markers could be used as potent predictive biomarkers associated with response to therapy and survival in cancer patients treated with immune checkpoint inhibitors. For example, circulating microRNAs (miRNAs) are able to alter the expression of PD-L1 on cancer cells, which may ultimately result in increased overall survival in cancer patients [118]. In a study by Halvorsen et al., a 7-miRNA signature (miR-215-5p, miR-411-3p, miR-493-5p, miR-494-3p, miR-495-3p, miR-548j-5p, and miR-93-3p) was associated with survival in nivolumab-treated NSCLC patients [119]. Some researchers have speculated that these miRNAs might induce cell cycle arrest or regulate interferon-*λ*1 [120, 121]. In another study, Shukuya and colleagues demonstrated that the concentration of specific circulating miRNAs and miRNAs packaged in extracellular vehicles was significantly different between responder and nonresponder NSCLC patients treated with anti-PD-1/L1 therapy [122]. Similarly, the expression profile of serum miRNAs was associated with response to therapy as well as survival in a study conducted on NSCLC patients receiving anti-PD-1 treatment [123]. miRNAs have also been investigated as predictive biomarkers in patients with malignant melanoma; Nakahara et al. found that the serum expression level of three miRNAs (miR-16-5p, miR-17-5p, and miR-20a-5p) was an independent predictor of response to nivolumab/pembrolizumab [124]. Altogether, circulating miRNAs could be introduced as useful predictors of efficacy of anti-PD-1/L1 immunotherapy given their stability and presence in biofluids. However, the exact mechanisms and target molecules of these miRNAs are not clearly understood.

### 8.2. Combination Therapies

In 2015, Mahoney et al. [14, 125] found that cancers that expressed PDL-1 receptors were more responsive to treatment with single-agent PD-1 blockers than tumors without PDL-1 expression. Tumor immune-cell infiltration is generally divided into three patterns: (i) “immune-desert” or noninflamed, (ii) “hot” or inflamed, and (iii) immune-excluded [126, 127]. Though T cell infiltration is not a definitive diagnostic/prognostic marker for PD-1 blocker activity, several researchers have reported a better response of highly infiltrated “hot” tumors to PD-1 blockers compared with other infiltration patterns [126, 128, 129]. The fact that anti-CTLA-4 agents improve T cell infiltration into the tumor microenvironment [130] provides an opportunity for PD-1 blockade agents to work more efficiently, hence, proving that combination therapy of the two agents is most superior.

Another approach that clinicians used to convert a cold tumor into a hot, inflamed tumor, which is more responsive to immunotherapy, was amalgamating PD-1 blockade with oncolytic viruses, such as the Newcastle virus [131]. Oncolytic virotherapy with talimogene laherparepvec increased cytotoxic T cell infiltration and therapeutic efficacy of the anti-PD-1 antibody, pembrolizumab, in advanced melanoma [105].

Combination therapy is a well-warranted tactic in cancer management [132]. The objective response rate, disease control response, and PFS all showed superiority in patients treated with combined chemotherapy and PD-1 blockade in comparison with either single therapies [131, 133]. Moreover, uniting local ablation and immunotherapies such as PD-1 inhibitors is one of the most potent regimens that oncologists can manipulate. One vital weakness of PD-1 blockers is their inability to penetrate the cancer microenvironment in cold tumors [134], which can be gained with the assistance of local ablation [135]. Hence, this combination will potentiate the effectiveness of both treatments, especially in solid tumors [136]. Many forms of local ablation can be combined with immune checkpoint inhibitors, including stereotactic body radiotherapy (SBRT), a method of killing tumor cells by damaging DNA, which makes the growing tumor cells more sensitive to radiation than normal tissues, and cryoablation, which destroys tumor tissue through several cycles of extremely cold temperatures and thawing [137]. These are relatively novel and minimally invasive local therapeutic options used for several solid tumors [136].

Moreover, studies have reported a synergistic combination of cancer vaccines and checkpoint blockers, in which cancer vaccines prime patients for PD-1 inhibitor therapy by inducing effector T cell infiltration into the tumors and immune checkpoint signals, basically turning “cold” tumors to “hot” T cell infiltrative tumors, improving the effectiveness of PD-1 blockers [132, 138].

### 8.3. Further Research

Currently, there is a need for improving the effectiveness of PD-1/L1 blockade by discovering novel predictive, diagnostic, and prognostic pharmacological biomarkers to make immunotherapy a more specific treatment with better clinical results and fewer adverse effects. Investigating new immunotherapeutic approaches that would inhibit a wider spectrum of inhibitory receptors, such as T cell immunoglobulin and mucin domain-containing protein 3 (TIM-3), lymphocyte-activation gene-3 (LAG-3), T cell immunoglobulin and ITIM domain (TIGIT), and B- and T-lymphocyte-associated protein (BTLA) receptors associated with T cell exhaustion as well as V-domain immunoglobulin suppressor of T cell activation (VISTA) is an achievable milestone [139], for instance, the inhibitory receptor VISTA on tumor-infiltrating myeloid cells, whose inhibition promotes antitumor immune responses in mice, and CD96, which is shown to inhibit NK cell activity in murine cancer models [12, 140].

In an effort to noninvasively monitor T cell infiltration within the tumor microenvironment and to predict response to treatment, novel radiolabeled tracers have been developed. Ongoing clinical trials are currently investigating the clinical utility of PD-1/L1-targeted positron emission tomography- (PET-) based imaging biomarkers, such as64Cu-WL12, 99mTc-NM-01, 89Zr-envafolimab, 18F-BMS-986192, 89Zr-durvalumab, 89Zr-labeled avelumab, 89Zr-labeled atezolizumab, 89Zr-CX-072, 89Zr-labeled atezolizumab, 89Zr-nivolumab, and 89Zr-pembrolizumab for detection of PD-1/L1 expression, T cell activation, or assessment of response to treatment in cancer patients [141, 142]. Recently, Niemeijer et al. showed that noninvasive evaluation of PD-1/L1 expression is feasible with PET-CT using the radiotracers 18F-BMS-986192 and 89Zr-nivolumab in patients with advanced NSCLC [143]. A phase II clinical trial is aimed at evaluating the correlation of uptake of an 89Zr-labeled anti-CD8 minibody (89Zr-labeled-IAB22M2C) with clinical response in patients with metastatic solid tumors treated with immunotherapy [144]. Although immunoPET imaging seems to show promise for optimizing individualized medicine in cancer immunotherapy, this approach faces many challenges. For example, the imaging tracers currently being used are not solely specific to T cells [142]. Another concern is that the T cells detected through immunoPET imaging may be anergic or exhausted, thereby not contributing to anti-tumor response [141]. Thus, the development of novel radiotracers that can be attributed more specifically to activated T cells may be essential in the future.

Recent advancements in the field of cellular and molecular immuno-oncology have revealed that cellular heterogeneity of TILs as well as the diverse and complex interactions between tumor and immune cells play a pivotal role in antitumor immune response and in response to cancer therapies including immune checkpoint blockade. Single-cell sequencing technologies are capable of providing a detailed characterization of individual immune cells, which may facilitate the efficacy of anticancer immunotherapies [145]. In a single-cell RNA sequencing study, Sehgal and colleagues found that a cancer cell subpopulation expressing Snai1 and Sca-1, namely, immunotherapy persister cells (IPCs), escaped CD8+ T cell-mediated killing after effective PD-1 blockade. They also showed that the combination of PD-1 blockade with Birc2/3 antagonists resulted in durable responses in mice through decreasing IPCs [146]. In another study, which used high-dimensional single-cell mass cytometry, the frequency of CD14+CD16−HLA-DRhi monocytes before treatment was found to be a predictor of response to anti-PD-1 immunotherapy in patients with melanoma [147]. Mass cytometry, also known as Cytometry by Time-Of-Flight (CyTOF), is an innovative next-generation flow cytometry platform that allows for high-dimensional phenotypic and functional analysis of single cells with several technological advantages over fluorescence-based flow cytometry. The platform enables the simultaneous quantification of over 40 parameters in individual cells with minimal overlap between channels [148, 149]. Better understanding of the tumor immune atlas through advanced single-cell technologies could revolutionize cancer immunotherapy and guide physicians in clinical practice.

Genomic approaches to predicting response or resistance to immunotherapy is an exciting strategy in the field of cancer immunology research. As mentioned earlier, studies have shown that tumor mutation burden is associated with response to anti-PD-1 immunotherapy in cancer patients. Incorporating high-resolution assays including single-cell gene expression profiling, T cell repertoire sequencing, whole-exome sequencing (WES), and targeted sequencing on circulating tumor cells (CTCs), cell-free DNA (cfDNA), and tumor tissue may shape the future of precision medicine by identification of genes in both tumors and T cells that are involved in response or resistance to cancer immunotherapy [150, 151]. In a recent study by Giroux Leprieur et al., WES performed on circulating tumor DNA (ctDNA) in advanced NSCLC patients showed that clonal selection with molecular alterations of Wnt pathway-related genes, increase of copy number aberrations in cancer-related genes, and loss of tumor-suppressor genes or genes associated with immune response seem to be associated with late progression under immune checkpoint inhibitor monotherapy [152]. Also, WES of NSCLC tumors treated with immune checkpoint inhibitors revealed that activating mutations in receptor tyrosine kinase (RTK) genes were significantly enriched in nonresponders [153]. In addition, decrease in tumor fraction (TFx) of cfDNA has been reported as an early biomarker of response to therapy in patients with castration-resistant prostate cancer [154]. However, the association between TFx decline and response to immune checkpoint blockade has not been studied yet. Single-cell T cell receptor (TCR) sequencing, which allows for sequencing of paired alpha and beta chains, is a novel but relatively expensive approach that may help in monitoring response to immunotherapies such as anti-PD-1/L1 immunotherapy [155, 156]. In a study sequencing the complementarity-determining region 3 (CDR3) of TCR*β* chains isolated from peripheral PD-1+ CD8+ T cells, high PD-1+ CD8+ TCR diversity before anti-PD-1/L1 immunotherapy was positively correlated with PFS and response to therapy in patients with NSCLC. Moreover, increased PD-1+ CD8+ TCR clonality after therapy was associated with longer PFS [157]. Similarly, a diverse baseline TCR repertoire was associated with a better response to anti-PD-1 therapy in patients with classical Hodgkin lymphoma (cHL). Also, CD4+ TCR diversity significantly increased during treatment, as cHL is a tumor with frequent major histocompatibility complex (MHC) class I loss [158].

Chimeric antigen receptor (CAR) T cell therapy is another effective and promising immunotherapy approach, which involves the genetic alteration of patients' T cells to express a CAR that is specific for a tumor antigen [159, 160]. This genetic modification may occur either via viral-based gene transfer methods or nonviral methods such as DNA-based transposons, clustered regularly interspaced short palindromic repeats/CRISPR associated protein 9(CRISPR/Cas9) technology, or direct transfer of in vitro transcribed-mRNA by electroporation. Clinical trials have shown promising results with CAR T cell therapy in end-stage patients with acute lymphocytic leukemia (ALL) with a full recovery of up to 92% [161]. This immunotherapeutic approach also holds much potential for osteosarcoma treatment [162]. The combination of CAR T cell therapy and PD-1 blockade is a highly potential strategy that enhances the therapeutic effects of CAR T cell therapy, especially in hematological cancers [163]. The pursuit of finding combination therapies should be further explored to establish combination-based clinical parameters, such as individual tolerability, dose, safety, durability, and efficacy [164]. It should be noted, however, that combination therapies carry the risk of toxicity to normal tissues expressing the same antigens as the tumor cells, and so, strategies are needed to reduce the on-target off-tumor toxicities [165].

Finally, the hallmark of the maximum clinical benefit of PD-1/L1 blockers in immunotherapy would be realized by combining them with other oncotherapies. Possible collaborators include other checkpoint inhibitors, kinase inhibitors, chemoradiotherapeutics, ablation, and cryoradiotherapies [166–168]. For example, combined oncolytic viral therapy and PD-1/L1 blockade have yielded favorable results with an objective response rate of 62% compared with 33% of PD-1/L1 monotherapy and a complete response rate of 33% per immune-related response criteria [105].

## 9. Conclusion

It is well-known that the PD-1/L1 pathway promotes cancer development and progression through negatively regulating T cell-mediated immune responses and suppressing proliferation, migration, and effector function of T cells. The introduction of PD-1/L1 blockade therapy has demonstrated promising antitumor effects in cancer immunotherapy of many different solid and hematologic malignancies. This form of immunotherapy is especially effective in tumors exhibiting high PDL-1/2 expression, MSI, TML, and dMMR; however, tumors not expressing any of these biomarkers have also responded well to treatment with immune checkpoint inhibitors. The use of multiple biomarkers is likely to be more effective for predicting responders than each of these biomarkers alone. In addition, defining an optimal cut-point of TMB for selecting patients with different cancer types is necessary. Developing novel drugs that block other coinhibitory receptors, such as TIM-3, LAG-3, TIGIT, BTLA, and VISTA, deserves more attention in the future. Finding novel strategies for converting “cold” to “hot” tumors with higher T cell infiltration in the tumor microenvironment will provide more desirable response rates with PD-1/L1 blockade therapy.

The sophisticated tumor immune microenvironment makes it difficult to utterly suppress the inhibitory microenvironment by a single-agent therapy. In this review, we discussed the benefits of combination therapy in terms of both reducing toxicities and increasing efficacy. A perfect example in this regard is the combination treatment of anti-CTLA-4 and anti-PD-1 antibodies, which has proved to be a feasible strategy significantly increasing overall response rate and demonstrating an acceptable safety profile compared with anti-PD-1 or anti-CTLA-4 monotherapy [58]. Other than anti-CTLA-4, several other interventions such as cancer vaccines, oncolytic viruses, and radiotherapy have been adopted to enhance the efficacy of PD-1 blockade therapy. One of the mechanisms of this enhanced efficacy is through increasing T cell infiltration in the tumor microenvironment. Considering the wide range of potential combinations, a deeper understanding of the cellular and molecular mechanisms of resistance and synergies will help improve the rational design of combination therapeutic strategies that can translate basic science to patient care. Taken together, the future landscape of these combination strategies seems to be very promising. To apply these novel strategies in the clinic, identifying more specific biomarkers is of utmost importance. There is still vast research that needs to be undertaken to rightfully select the most appropriate cancers which deserve to be treated with PD-1/L-1/2 blockers as immunotherapy. Furthermore, the identification of dynamic rather than static biomarkers that could predict response or resistance to anti-PD-1/L1 immunotherapy will have a significant impact in routine clinical management of cancer patients.

## Figures and Tables

**Table 1:** Comparison of survival probability with various regimens for advanced melanoma after 12 months of treatment.

Treatment regimen	Survival probability (%)	Reference
Dacarbazine	12	[40]
Dabrafenib + trametinib	46	[41]
Ipilimumab	46	[42]
Nivolumab	72.9	[42]
Pembrolizumab	74.1	[42]
Vemurafenib+ cobimetinib	73	[43]

**Table 2:** FDA-approved PD-1/L1/2 inhibitors for various malignancies.

Target	Agent	Class	Cancer
PD-1	Pembrolizumab (MK-3475, Keytruda)	Humanized IgG4k	Melanoma
			Breast cancer
			Non-small-cell lung cancer
			Small-cell lung cancer
			Head and neck squamous cell cancer
			Hodgkin lymphoma
			Gastric/gastroesophageal junction (GEJ) adenocarcinoma
			Cervical cancer
			Endometrial carcinoma
			Primary mediastinal large B cell lymphoma
			Hepatocellular carcinoma
			Merkel cell carcinoma
			Renal cell cancer
			Urothelial cancer
	Nivolumab (MDX1106, Opdivo)	Humanized IgG4	Melanoma
			Non-small-cell lung cancer
			Small-cell lung cancer
			Renal cell cancer
			Hodgkin lymphoma
			Head and neck squamous cell cancer
			Urothelial cancer
			Colorectal cancer
			Hepatocellular carcinoma
			Metastatic esophageal squamous cell carcinoma (ESCC)
	Cemiplimab (Libtayo)	Monoclonal antibody/antibody-drug conjugate	Cutaneous squamous cell carcinoma

PD-L1	Atezolizumab (MPDL-3280A, Tecentriq)	Humanized IgG1k	Urothelial carcinoma
			Non-small-cell lung cancer
			Small-cell lung cancer
			Hepatocellular carcinoma
			Breast cancer
	Durvalumab (MEDI4736, Imfinzi)	Human IgG1k	Urothelial carcinoma
			Non-small-cell lung cancer
			Small-cell lung cancer
	Avelumab (MSB0010718C, Bavencio)	Human IgG1	Merkel cell carcinoma
			Urothelial carcinoma
			Renal cell cancer

Data are included as of June 5, 2020. Ig: immunoglobulin; PD-1: programmed cell death protein 1; PD-L1: programmed death ligand 1.

## Data Availability

No data were used to support this study.

## References

[1] Nagai H., Kim Y. H. (2017). Cancer prevention from the perspective of global cancer burden patterns. *Journal of Thoracic Disease*.

[2] Dimberu P. M., Leonhardt R. M. (2011). Cancer immunotherapy takes a multi-faceted approach to kick the immune system into gear. *The Yale Journal of Biology and Medicine*.

[3] Wraith D. C. (2017). The future of immunotherapy: a 20-year perspective. *Frontiers in Immunology*.

[4] Alsaab H. O., Sau S., Alzhrani R. (2017). PD-1 and PD-L1 checkpoint signaling inhibition for cancer immunotherapy: mechanism, combinations, and clinical outcome. *Frontiers in Pharmacology*.

[5] Bonanno L., Pavan A., Attili I., Pasello G., Guarneri V. (2019). Immunotherapy in SCLC: exceptional clinical benefit and abscopal pneumonitis after radiotherapy. *Journal of Thoracic Oncology*.

[6] Ishida Y., Agata Y., Shibahara K., Honjo T. (1992). Induced expression of PD-1, a novel member of the immunoglobulin gene superfamily, upon programmed cell death. *The EMBO Journal*.

[7] Nishimura H., Nose M., Hiai H., Minato N., Honjo T. (1999). Development of lupus-like autoimmune diseases by disruption of the _PD-1_ gene encoding an ITIM motif-carrying immunoreceptor. *Immunity*.

[8] Nishimura H., Okazaki T., Tanaka Y. (2001). Autoimmune dilated cardiomyopathy in PD-1 receptor-deficient mice. *Science (New York, NY)*.

[9] Gong J., Chehrazi-Raffle A., Reddi S., Salgia R. (2018). Development of PD-1 and PD-L1 inhibitors as a form of cancer immunotherapy: a comprehensive review of registration trials and future considerations. *Journal for ImmunoTherapy of Cancer*.

[10] Food and Drug Administration (FDA) (2020). FDA approves pembrolizumab for BCG-unresponsive, high-risk non-muscle invasive bladder cancer. https://www.fda.gov/drugs/resources-information-approved-drugs/fda-approves-pembrolizumab-bcg-unresponsive-high-risk-non-muscle-invasive-bladder-cancer.

[11] Food and Drug Administration (FDA) (2020). FDA approves nivolumab for esophageal squamous cell carcinoma. https://www.fda.gov/drugs/drug-approvals-and-databases/fda-approves-nivolumab-esophageal-squamous-cell-carcinoma.

[12] Seidel J. A., Otsuka A., Kabashima K. (2018). Anti-PD-1 and anti-CTLA-4 therapies in cancer: mechanisms of action, efficacy, and limitations. *Frontiers in Oncology*.

[13] Granier C., De Guillebon E., Blanc C. (2017). Mechanisms of action and rationale for the use of checkpoint inhibitors in cancer. *ESMO Open*.

[14] Mahoney K. M., Freeman G. J., McDermott D. F. (2015). The next immune-checkpoint inhibitors: PD-1/PD-L1 blockade in melanoma. *Clinical Therapeutics*.

[15] Francisco L. M., Sage P. T., Sharpe A. H. (2010). The PD-1 pathway in tolerance and autoimmunity. *Immunological Reviews*.

[16] Ostrand-Rosenberg S., Horn L. A., Haile S. T. (2014). The programmed death-1 immune-suppressive pathway: barrier to antitumor immunity. *Journal of Immunology (Baltimore, Md : 1950)*.

[17] Fife B. T., Pauken K. E. (2011). The role of the PD-1 pathway in autoimmunity and peripheral tolerance. *Annals of the New York Academy of Sciences*.

[18] Rozali E. N., Hato S. V., Robinson B. W., Lake R. A., Lesterhuis W. J. (2012). Programmed death ligand 2 in cancer-induced immune suppression. *Clinical and Developmental Immunology*.

[19] Wang B., Zhang W., Jankovic V. (2018). Combination cancer immunotherapy targeting PD-1 and GITR can rescue CD8(+) T cell dysfunction and maintain memory phenotype. *Science Immunology*.

[20] Butte M. J., Keir M. E., Phamduy T. B., Sharpe A. H., Freeman G. J. (2007). Programmed death-1 ligand 1 interacts specifically with the B7-1 costimulatory molecule to inhibit T cell responses. *Immunity*.

[21] Escors D., Gato-Cañas M., Zuazo M. (2018). The intracellular signalosome of PD-L1 in cancer cells. *Signal Transduction and Targeted Therapy*.

[22] Yi M., Jiao D., Xu H. (2018). Biomarkers for predicting efficacy of PD-1/PD-L1 inhibitors. *Molecular Cancer*.

[23] Bai J., Gao Z., Li X., Dong L., Han W., Nie J. (2017). Regulation of PD-1/PD-L1 pathway and resistance to PD-1/PD-L1 blockade. *Oncotarget*.

[24] Jelinek T., Paiva B., Hajek R. (2018). Update on PD-1/PD-L1 inhibitors in multiple myeloma. *Frontiers in Immunology*.

[25] Brunner-Weinzierl M. C., Rudd C. E. (2018). CTLA-4 and PD-1 control of T-cell motility and migration: implications for tumor immunotherapy. *Frontiers in Immunology*.

[26] Kamphorst A. O., Pillai R. N., Yang S. (2017). Proliferation of PD-1+ CD8 T cells in peripheral blood after PD-1–targeted therapy in lung cancer patients. *Proceedings of the National Academy of Sciences*.

[27] Dermani F. K., Samadi P., Rahmani G., Kohlan A. K., Najafi R. (2019). PD-1/PD-L1 immune checkpoint: potential target for cancer therapy. *Journal of Cellular Physiology*.

[28] Sheppard K.-A., Fitz L. J., Lee J. M. (2004). PD-1 inhibits T-cell receptor induced phosphorylation of the ZAP70/CD3*ζ* signalosome and downstream signaling to PKC*θ*. *FEBS Letters*.

[29] Sitaram P., Uyemura B., Malarkannan S., Riese M. J. (2019). Beyond the cell surface: targeting intracellular negative regulators to enhance T cell anti-tumor activity. *International Journal of Molecular Sciences*.

[30] Riley J. L. (2009). PD-1 signaling in primary T cells. *Immunological Reviews*.

[31] McCubrey J. A., Steelman L. S., Chappell W. H. (2007). Roles of the Raf/MEK/ERK pathway in cell growth, malignant transformation and drug resistance. *Biochimica et Biophysica Acta (BBA) - Molecular Cell Research*.

[32] Shi X., Wang J., Lei Y., Cong C., Tan D., Zhou X. (2019). Research progress on the PI3K/AKT signaling pathway in gynecological cancer (Review). *Molecular Medicine Reports*.

[33] Vara J. Á. F., Casado E., de Castro J., Cejas P., Belda-Iniesta C., González-Barón M. (2004). PI3K/Akt signalling pathway and cancer. *Cancer Treatment Reviews*.

[34] Zhao R., Song Y., Wang Y. (2019). PD-1/PD-L1 blockade rescue exhausted CD8+ T cells in gastrointestinal stromal tumours via the PI3K/Akt/mTOR signalling pathway. *Cell Proliferation*.

[35] Cretella D., Digiacomo G., Giovannetti E., Cavazzoni A. (2019). PTEN alterations as a potential mechanism for tumor cell escape from PD-1/PD-L1 inhibition. *Cancers (Basel)*.

[36] Pompura S. L., Dominguez-Villar M. (2018). The PI3K/AKT signaling pathway in regulatory T-cell development, stability, and function. *Journal of Leukocyte Biology*.

[37] Webb E. S., Liu P., Baleeiro R., Lemoine N. R., Yuan M., Wang Y. (2018). Immune checkpoint inhibitors in cancer therapy. *Journal of Biomedical Research*.

[38] Sasidharan Nair V., Elkord E. (2018). Immune checkpoint inhibitors in cancer therapy: a focus on T-regulatory cells. *Immunology and Cell Biology*.

[39] Jazirehi A. R., Lim A., Dinh T. (2016). PD-1 inhibition and treatment of advanced melanoma-role of pembrolizumab. *American Journal of Cancer Research*.

[40] Bhatia S., Tykodi S. S., Lee S. M., Thompson J. A. (2015). Systemic therapy of metastatic melanoma: on the road to cure. *Oncology*.

[41] Davies M. A., Saiag P., Robert C. (2017). Dabrafenib plus trametinib in patients with _BRAF_ ^V600^-mutant melanoma brain metastases (COMBI-MB): a multicentre, multicohort, open-label, phase 2 trial. *The Lancet Oncology*.

[42] Lugowska I., Teterycz P., Rutkowski P. (2018). Immunotherapy of melanoma. *Contemporary Oncology*.

[43] Larkin J., Ascierto P. A., Dréno B. (2014). Combined vemurafenib and cobimetinib in BRAF-mutated melanoma. *The New England Journal of Medicine*.

[44] Robert C., Schachter J., Long G. V. (2015). Pembrolizumab versus ipilimumab in advanced melanoma. *New England Journal of Medicine*.

[45] Stenehjem D. D., Tran D., Nkrumah M. A., Gupta S. (2018). PD1/PDL1 inhibitors for the treatment of advanced urothelial bladder cancer. *OncoTargets and Therapy*.

[46] Sgambato A., Casaluce F., Sacco P. C. (2016). Anti PD-1 and PDL-1 immunotherapy in the treatment of advanced non-small cell lung cancer (NSCLC): a review on toxicity profile and its management. *Current Drug Safety*.

[47] Teng M. W., Ngiow S. F., Ribas A., Smyth M. J. (2015). Classifying cancers based on T-cell infiltration and PD-L1. *Cancer Research*.

[48] Khan M., Lin J., Liao G. (2018). Comparative analysis of immune checkpoint inhibitors and chemotherapy in the treatment of advanced non-small cell lung cancer: a meta-analysis of randomized controlled trials. *Medicine*.

[49] Kopetz S., Lonardi S., McDermott R. S. (2017). Concordance of DNA mismatch repair deficient (dMMR)/microsatellite instability (MSI) assessment by local and central testing in patients with metastatic CRC (mCRC) receiving nivolumab (nivo) in CheckMate 142 study. *American Society of Clinical Oncology*.

[50] Le DT U. J. N., Wang H., Bartlett B. R. (2015). PD-1 blockade in tumors with mismatch-repair deficiency. *New England Journal of Medicine*.

[51] Salem M. E., Puccini A., Grothey A. (2018). Landscape of tumor mutation load, mismatch repair deficiency, and PD-L1 expression in a large patient cohort of gastrointestinal cancers. *Molecular Cancer Research*.

[52] Yarchoan M., Hopkins A., Jaffee E. M. (2017). Tumor mutational burden and response rate to PD-1 inhibition. *The New England Journal of Medicine*.

[53] Gandara D. R., Paul S. M., Kowanetz M. (2018). Blood-based tumor mutational burden as a predictor of clinical benefit in non-small-cell lung cancer patients treated with atezolizumab. *Nature Medicine*.

[54] Wu X., Gu Z., Chen Y. (2019). Application of PD-1 blockade in cancer immunotherapy. *Computational and Structural Biotechnology Journal*.

[55] Pike E., Hamidi V., Saeterdal I., Odgaard-Jensen J., Klemp M. (2017). Multiple treatment comparison of seven new drugs for patients with advanced malignant melanoma: a systematic review and health economic decision model in a Norwegian setting. *BMJ Open*.

[56] Burns M. C., O'Donnell A., Puzanov I. (2016). Pembrolizumab for the treatment of advanced melanoma. *Expert Opinion on Orphan Drugs*.

[57] Domingues B., Lopes J. M., Soares P., Pópulo H. (2018). Melanoma treatment in review. *ImmunoTargets and Therapy*.

[58] Wu K., Yi M., Qin S., Chu Q., Zheng X., Wu K. (2019). The efficacy and safety of combination of PD-1 and CTLA-4 inhibitors: a meta-analysis. *Experimental Hematology & Oncology*.

[59] Constantinidou A., Alifieris C., Trafalis D. T. (2019). Targeting programmed cell death -1 (PD-1) and ligand (PD-L1): a new era in cancer active immunotherapy. *Pharmacology & Therapeutics*.

[60] Gubin M. M., Zhang X., Schuster H. (2014). Checkpoint blockade cancer immunotherapy targets tumour-specific mutant antigens. *Nature*.

[61] Xu C., Chen Y.-P., Du X.-J. (2018). Comparative safety of immune checkpoint inhibitors in cancer: systematic review and network meta-analysis. *BMJ*.

[62] Schuetz F., Ehlert K., Ge Y. (2009). Treatment of advanced metastasized breast cancer with bone marrow-derived tumour-reactive memory T cells: a pilot clinical study. *Cancer Immunology, Immunotherapy*.

[63] Domschke C., Ge Y., Bernhardt I. (2013). Long-term survival after adoptive bone marrow T cell therapy of advanced metastasized breast cancer: follow-up analysis of a clinical pilot trial. *Cancer Immunology, Immunotherapy*.

[64] Mall C., Sckisel G. D., Proia D. A. (2015). Repeated PD-1/PD-L1 monoclonal antibody administration induces fatal xenogeneic hypersensitivity reactions in a murine model of breast cancer. *Oncoimmunology*.

[65] Hofmann L., Forschner A., Loquai C. (2016). Cutaneous, gastrointestinal, hepatic, endocrine, and renal side-effects of anti-PD-1 therapy. *European Journal of Cancer (Oxford, England : 1990)*.

[66] Naidoo J., Page D. B., Li B. T. (2015). Toxicities of the anti-PD-1 and anti-PD-L1 immune checkpoint antibodies. *Annals of Oncology*.

[67] Kennedy L. B., Salama A. K. S. (2020). A review of cancer immunotherapy toxicity. *CA: A Cancer Journal for Clinicians*.

[68] Nishino M., Sholl L. M., Hatabu H., Ramaiya N. H., Hodi F. S. (2015). Anti-PD-1-related pneumonitis during cancer immunotherapy. *The New England Journal of Medicine*.

[69] Wu J., Hong D., Zhang X., Lu X., Miao J. (2017). PD-1 inhibitors increase the incidence and risk of pneumonitis in cancer patients in a dose-independent manner: a meta-analysis. *Scientific Reports*.

[70] Naidoo J., Wang X., Woo K. M. (2017). Pneumonitis in patients treated with anti-programmed death-1/programmed death ligand 1 therapy. *Journal of Clinical Oncology*.

[71] Su Q., Zhu E. C., Wu J.-b. (2019). Risk of pneumonitis and pneumonia associated with immune checkpoint inhibitors for solid tumors: a systematic review and meta-analysis. *Frontiers in Immunology*.

[72] Kähler K. C., Hassel J. C., Heinzerling L. (2016). Management of side effects of immune checkpoint blockade by anti-CTLA-4 and anti-PD-1 antibodies in metastatic melanoma. *JDDG: Journal der Deutschen Dermatologischen Gesellschaft*.

[73] Läubli H., Balmelli C., Bossard M., Pfister O., Glatz K., Zippelius A. (2015). Acute heart failure due to autoimmune myocarditis under pembrolizumab treatment for metastatic melanoma. *Journal for Immunotherapy of Cancer*.

[74] Nowicki T. S., Hu-Lieskovan S., Ribas A. (2018). Mechanisms of resistance to PD-1 and PD-L1 blockade. *Cancer Journal*.

[75] Klemen N. D., Wang M., Feingold P. L. (2019). Patterns of failure after immunotherapy with checkpoint inhibitors predict durable progression-free survival after local therapy for metastatic melanoma. *Journal for ImmunoTherapy of Cancer*.

[76] Saâda-Bouzid E., Defaucheux C., Karabajakian A. (2017). Hyperprogression during anti-PD-1/PD-L1 therapy in patients with recurrent and/or metastatic head and neck squamous cell carcinoma. *Annals of Oncology*.

[77] Kim C. G., Kim K. H., Pyo K. H. (2019). Hyperprogressive disease during PD-1/PD-L1 blockade in patients with non-small-cell lung cancer. *Annals of Oncology*.

[78] Johnson D. B., Sullivan R. J., Menzies A. M. (2017). Immune checkpoint inhibitors in challenging populations. *Cancer*.

[79] Meng X., Huang Z., Teng F., Xing L., Yu J. (2015). Predictive biomarkers in PD-1/PD-L1 checkpoint blockade immunotherapy. *Cancer Treatment Reviews*.

[80] Shen X., Zhao B. (2018). Efficacy of PD-1 or PD-L1 inhibitors and PD-L1 expression status in cancer: meta-analysis. *BMJ*.

[81] Chen Q., Li T., Yue W. (2018). Drug response to PD-1/PD-L1 blockade: based on biomarkers. *OncoTargets and Therapy*.

[82] Udall M., Rizzo M., Kenny J. (2018). PD-L1 diagnostic tests: a systematic literature review of scoring algorithms and test-validation metrics. *Diagnostic Pathology*.

[83] Kawakami H., Zaanan A., Sinicrope F. A. (2015). Microsatellite instability testing and its role in the management of colorectal cancer. *Current Treatment Options in Oncology*.

[84] Abida W., Cheng M. L., Armenia J. (2019). Analysis of the prevalence of microsatellite instability in prostate cancer and response to immune checkpoint blockade. *JAMA Oncology*.

[85] Yarchoan M., Albacker L. A., Hopkins A. C. (2019). PD-L1 expression and tumor mutational burden are independent biomarkers in most cancers. *JCI Insight*.

[86] Danilova L., Wang H., Sunshine J. (2016). Association of PD-1/PD-L axis expression with cytolytic activity, mutational load, and prognosis in melanoma and other solid tumors. *Proceedings of the National Academy of Sciences of the United States of America*.

[87] Budczies J., Mechtersheimer G., Denkert C. (2017). PD-L1 (CD274) copy number gain, expression, and immune cell infiltration as candidate predictors for response to immune checkpoint inhibitors in soft-tissue sarcoma. *Oncoimmunology*.

[88] Budczies J., Denkert C., Győrffy B., Schirmacher P., Stenzinger A. (2017). Chromosome 9p copy number gains involving PD-L1 are associated with a specific proliferation and immune-modulating gene expression program active across major cancer types. *BMC Medical Genomics*.

[89] Ansell S. M., Lesokhin A. M., Borrello I. (2014). PD-1 blockade with nivolumab in relapsed or refractory Hodgkin’s lymphoma. *New England Journal of Medicine*.

[90] Lee H. H., Wang Y. N., Xia W. (2019). Removal of N-linked glycosylation enhances PD-L1 detection and predicts anti-PD-1/PD-L1 therapeutic efficacy. *Cancer Cell*.

[91] Kye S. Y., Park E. Y., Oh K., Park K. (2015). Perceptions of cancer risk and cause of cancer risk in Korean adults. *Cancer Research and Treatment*.

[92] Kelly Z. R., Davar D. (2019). *The Financial and Physical Toxicity of Immune Checkpoint Inhibitors in Cancer Alexandria*.

[93] Aguiar P. N., Perry L. A., Penny-Dimri J. (2017). The effect of PD-L1 testing on the cost-effectiveness and economic impact of immune checkpoint inhibitors for the second-line treatment of NSCLC. *Annals of Oncology*.

[94] Wood P. (2012). Immunotherapy for primary immunodeficiency diseases. *The Medical Clinics of North America*.

[95] Marciano B. E., Holland S. M. (2017). Primary immunodeficiency diseases: current and emerging therapeutics. *Frontiers in Immunology*.

[96] Nowak-Węgrzyn A., Albin S. (2015). Oral immunotherapy for food allergy: mechanisms and role in management. *Clinical and Experimental Allergy*.

[97] Michot J. M., Bigenwald C., Champiat S. (2016). Immune-related adverse events with immune checkpoint blockade: a comprehensive review. *European Journal of Cancer (Oxford, England : 1990)*.

[98] Tison A., Quéré G., Misery L. (2019). Safety and efficacy of immune checkpoint inhibitors in patients with cancer and preexisting autoimmune disease: a nationwide, multicenter cohort study. *Arthritis & Rheumatology*.

[99] Abdel-Wahab N., Shah M., Lopez-Olivo M. A., Suarez-Almazor M. E. (2018). Use of immune checkpoint inhibitors in the treatment of patients with cancer and preexisting autoimmune disease: a systematic review. *Annals of Internal Medicine*.

[100] Myers G. (2018). Immune-related adverse events of immune checkpoint inhibitors: a brief review. *Current Oncology*.

[101] Song M., Chen X., Wang L., Zhang Y. (2018). Future of anti-PD-1/PD-L1 applications: combinations with other therapeutic regimens. *Chinese Journal of Cancer Research = Chung-Kuo yen Cheng yen Chiu*.

[102] LaFleur M. W., Muroyama Y., Drake C. G., Sharpe A. H. (2018). Inhibitors of the PD-1 pathway in tumor therapy. *The Journal of Immunology*.

[103] Ramos R. N., Piaggio E., Romano E. (2017). Mechanisms of resistance to immune checkpoint antibodies. *Mechanisms of Drug Resistance in Cancer Therapy*.

[104] Koyama S., Akbay E. A., Li Y. Y. (2016). Adaptive resistance to therapeutic PD-1 blockade is associated with upregulation of alternative immune checkpoints. *Nature Communications*.

[105] Ribas A., Dummer R., Puzanov I. (2017). Oncolytic virotherapy promotes intratumoral T cell infiltration and improves anti-PD-1 immunotherapy. *Cell*.

[106] Higgs B. W., Morehouse C. A., Streicher K. (2018). Interferon gamma messenger RNA signature in tumor biopsies predicts outcomes in patients with non-small cell lung carcinoma or urothelial cancer treated with durvalumab. *Clinical Cancer Research*.

[107] Rizvi N. A., Hellmann M. D., Snyder A. (2015). Mutational landscape determines sensitivity to PD-1 blockade in non-small cell lung cancer. *Science (New York, NY)*.

[108] Routy B., Le Chatelier E., Derosa L. (2018). Gut microbiome influences efficacy of PD-1-based immunotherapy against epithelial tumors. *Science (New York, NY)*.

[109] Zheng Y., Wang T., Tu X. (2019). Gut microbiome affects the response to anti-PD-1 immunotherapy in patients with hepatocellular carcinoma. *Journal for ImmunoTherapy of Cancer*.

[110] Gopalakrishnan V., Spencer C. N., Nezi L. (2018). Gut microbiome modulates response to anti-PD-1 immunotherapy in melanoma patients. *Science (New York, NY)*.

[111] Elkrief A., Derosa L., Zitvogel L., Kroemer G., Routy B. (2019). The intimate relationship between gut microbiota and cancer immunotherapy. *Gut Microbes*.

[112] Zaragoza J., Caille A., Beneton N. (2016). High neutrophil to lymphocyte ratio measured before starting ipilimumab treatment is associated with reduced overall survival in patients with melanoma. *British Journal of Dermatology*.

[113] Capone M., Giannarelli D., Mallardo D. (2018). Baseline neutrophil-to-lymphocyte ratio (NLR) and derived NLR could predict overall survival in patients with advanced melanoma treated with nivolumab. *Journal for ImmunoTherapy of Cancer*.

[114] Xie X., Liu J., Yang H. (2019). Prognostic value of baseline neutrophil-to-lymphocyte ratio in outcome of immune checkpoint inhibitors. *Cancer Investigation*.

[115] Martens A., Wistuba-Hamprecht K., Foppen M. G. (2016). Baseline peripheral blood biomarkers associated with clinical outcome of advanced melanoma patients treated with ipilimumab. *Clinical Cancer Research*.

[116] Tomela K., Pietrzak B., Schmidt M., Mackiewicz A. (2020). The tumor and host immune signature, and the gut microbiota as predictive biomarkers for immune checkpoint inhibitor response in melanoma patients. *Life*.

[117] Prelaj A., Tay R., Ferrara R., Chaput N., Besse B., Califano R. (2019). Predictive biomarkers of response for immune checkpoint inhibitors in non- small-cell lung cancer. *European Journal of Cancer*.

[118] Li X., Warren S., Pelekanou V. (2019). Immune profiling of pre- and post-treatment breast cancer tissues from the SWOG S0800 neoadjuvant trial. *Journal for Immunotherapy of Cancer*.

[119] Halvorsen A. R., Sandhu V., Sprauten M. (2018). Circulating microRNAs associated with prolonged overall survival in lung cancer patients treated with nivolumab. *Acta Oncologica (Stockholm, Sweden)*.

[120] Li Y., Xie J., Xu X. (2013). MicroRNA-548 down-regulates host antiviral response via direct targeting of IFN-*λ*1. *Protein & Cell*.

[121] Georges S. A., Biery M. C., Kim S. (2008). Coordinated regulation of cell cycle transcripts by p53-inducible microRNAs, miR-192 and miR-215. *Cancer Research*.

[122] Shukuya T., Ghai V., Amann J. M. (2020). Circulating microRNAs and extracellular vesicle-containing MicroRNAs as response biomarkers of anti-programmed cell death protein 1 or programmed death-ligand 1 therapy in NSCLC. *Journal of Thoracic Oncology*.

[123] Fan J., Yin Z., Xu J. (2020). Circulating microRNAs predict the response to anti-PD-1 therapy in non-small cell lung cancer. *Genomics*.

[124] Nakahara S., Fukushima S., Okada E. (2020). MicroRNAs that predict the effectiveness of anti-PD-1 therapies in patients with advanced melanoma. *Journal of Dermatological Science*.

[125] Mahoney K. M., Rennert P. D., Freeman G. J. (2015). Combination cancer immunotherapy and new immunomodulatory targets. *Nature Reviews Drug Discovery*.

[126] Danaher P., Warren S., Lu R. (2018). Pan-cancer adaptive immune resistance as defined by the tumor inflammation signature (TIS): results from the cancer genome atlas (TCGA). *Journal for ImmunoTherapy of Cancer*.

[127] Kather J. N., Suarez-Carmona M., Charoentong P. (2018). Topography of cancer-associated immune cells in human solid tumors. *eLife*.

[128] Taube J. M., Galon J., Sholl L. M. (2018). Implications of the tumor immune microenvironment for staging and therapeutics. *Modern Pathology*.

[129] Cottrell T. R., Thompson E. D., Forde P. M. (2018). Pathologic features of response to neoadjuvant anti-PD-1 in resected non-small-cell lung carcinoma: a proposal for quantitative immune-related pathologic response criteria (irPRC). *Annals of Oncology*.

[130] Lanitis E., Dangaj D., Irving M., Coukos G. (2017). Mechanisms regulating T-cell infiltration and activity in solid tumors. *Annals of Oncology*.

[131] Shen K., Cui J., Wei Y. (2018). Effectiveness and safety of PD-1/PD-L1 or CTLA4 inhibitors combined with chemotherapy as a first-line treatment for lung cancer: a meta-analysis. *Journal of Thoracic Disease*.

[132] Kleponis J., Skelton R., Zheng L. (2015). Fueling the engine and releasing the break: combinational therapy of cancer vaccines and immune checkpoint inhibitors. *Cancer Biology & Medicine*.

[133] Schmid P., Cortes J., Pusztai L. (2020). Pembrolizumab for early triple-negative breast cancer. *New England Journal of Medicine*.

[134] Tang H., Qiao J., Fu Y. X. (2016). Immunotherapy and tumor microenvironment. *Cancer Letters*.

[135] Takahashi Y., Matsutani N., Nakayama T., Dejima H., Uehara H., Kawamura M. (2017). Immunological effect of local ablation combined with immunotherapy on solid malignancies. *Chinese Journal of Cancer*.

[136] Kyi C., Postow M. A. (2016). Immune checkpoint inhibitor combinations in solid tumors: opportunities and challenges. *Immunotherapy*.

[137] Aarts B. M., Klompenhouwer E. G., Rice S. L. (2019). Cryoablation and immunotherapy: an overview of evidence on its synergy. *Insights into Imaging*.

[138] Antonios J. P., Soto H., Everson R. G. (2016). PD-1 blockade enhances the vaccination-induced immune response in glioma. *JCI Insight*.

[139] Joller N., Kuchroo V. K. (2017). Tim-3, Lag-3, and TIGIT. *Emerging Concepts Targeting Immune Checkpoints in Cancer and Autoimmunity*.

[140] Sharma P., Hu-Lieskovan S., Wargo J. A., Ribas A. (2017). Primary, adaptive, and acquired resistance to cancer immunotherapy. *Cell*.

[141] Abousaway O., Rakhshandehroo T., Van den Abbeele A. D., Kircher M. F., Rashidian M. (2021). Noninvasive imaging of cancer immunotherapy. *Nano*.

[142] Wei W., Jiang D., Ehlerding E. B., Luo Q., Cai W. (2018). Noninvasive PET imaging of T cells. *Trends in Cancer*.

[143] Niemeijer A. N., Leung D., Huisman M. C. (2018). Whole body PD-1 and PD-L1 positron emission tomography in patients with non- small-cell lung cancer. *Nature Communications*.

[144] (2021). ^89^Zr-Df-IAB22M2C (CD8 PET Tracer) for PET/CT in patients with metastatic solid tumors. https://clinicaltrials.gov/ct2/show/NCT03802123.

[145] Zhang L., Zhang Z. (2019). Recharacterizing tumor-infiltrating lymphocytes by single-cell RNA sequencing. *Cancer Immunology Research*.

[146] Sehgal K., Portell A., Ivanova E. V. (2020). Dynamic single-cell RNA sequencing identifies immunotherapy persister cells following PD-1 blockade. *The Journal of Clinical Investigation*.

[147] Krieg C., Nowicka M., Guglietta S. (2018). High-dimensional single-cell analysis predicts response to anti-PD-1 immunotherapy. *Nature Medicine*.

[148] Kay A. W., Strauss-Albee D. M., Blish C. A. (2016). Application of mass cytometry (CyTOF) for functional and phenotypic analysis of natural killer cells. *Methods in Molecular Biology*.

[149] Spitzer M. H., Nolan G. P. (2016). Mass cytometry: single cells, many features. *Cell*.

[150] Hellmann M. D., Nathanson T., Rizvi H. (2018). Genomic features of response to combination immunotherapy in patients with advanced non-small-cell lung cancer. *Cancer Cell*.

[151] Braun D. A., Burke K. P., Van Allen E. M. (2016). Genomic approaches to understanding response and resistance to immunotherapy. *Clinical Cancer Research*.

[152] Giroux Leprieur E., Hélias-Rodzewicz Z., Takam Kamga P. (2020). Sequential ctDNA whole-exome sequencing in advanced lung adenocarcinoma with initial durable tumor response on immune checkpoint inhibitor and late progression. *Journal for Immunotherapy of Cancer*.

[153] Anagnostou V., Niknafs N., Marrone K. (2020). Multimodal genomic features predict outcome of immune checkpoint blockade in non-small-cell lung cancer. *Nature Cancer*.

[154] Choudhury A. D., Werner L., Francini E. (2018). Tumor fraction in cell-free DNA as a biomarker in prostate cancer. *JCI Insight*.

[155] Rosati E., Dowds C. M., Liaskou E., Henriksen E. K. K., Karlsen T. H., Franke A. (2017). Overview of methodologies for T-cell receptor repertoire analysis. *BMC Biotechnology*.

[156] De Simone M., Rossetti G., Pagani M. (2018). Single cell T cell receptor sequencing: techniques and future challenges. *Frontiers in Immunology*.

[157] Han J., Duan J., Bai H. (2020). TCR repertoire diversity of peripheral PD-1+ CD8+ T cells predicts clinical outcomes after immunotherapy in patients with non–small cell lung cancer. *Cancer Immunology Research*.

[158] Cader F. Z., Hu X., Goh W. L. (2020). A peripheral immune signature of responsiveness to PD-1 blockade in patients with classical Hodgkin lymphoma. *Nature Medicine*.

[159] Liu B., Song Y., Liu D. (2017). Clinical trials of CAR-T cells in China. *Journal of Hematology & Oncology*.

[160] Graham C., Hewitson R., Pagliuca A., Benjamin R. (2018). Cancer immunotherapy with CAR-T cells - behold the future. *Clinical medicine (London, England)*.

[161] Miliotou A. N., Papadopoulou L. C. (2018). CAR T-cell therapy: a new era in cancer immunotherapy. *Current Pharmaceutical Biotechnology*.

[162] Weibo P., Zhaoming Y. (2012). Auto T cells expressing chimeric antigen receptor derived from auto antibody might be a new treatment for osteosarcoma. *Medical Hypotheses*.

[163] Wang H., Kaur G., Sankin A. I., Chen F., Guan F., Zang X. (2019). Immune checkpoint blockade and CAR-T cell therapy in hematologic malignancies. *Journal of Hematology & Oncology*.

[164] Wang D., Lin J., Yang X. (2019). Combination regimens with PD-1/PD-L1 immune checkpoint inhibitors for gastrointestinal malignancies. *Journal of Hematology & Oncology*.

[165] Grosser R., Cherkassky L., Chintala N., Adusumilli P. S. (2019). Combination immunotherapy with CAR T cells and checkpoint blockade for the treatment of solid tumors. *Cancer Cell*.

[166] Kyi C., Hellmann M. D., Wolchok J. D., Chapman P. B., Postow M. A. (2014). Opportunistic infections in patients treated with immunotherapy for cancer. *Journal for Immunotherapy of Cancer*.

[167] Curran M. A., Montalvo W., Yagita H., Allison J. P. (2010). PD-1 and CTLA-4 combination blockade expands infiltrating T cells and reduces regulatory T and myeloid cells within B16 melanoma tumors. *Proceedings of the National Academy of Sciences of the United States of America*.

[168] Ott P. A., Hodi F. S., Robert C. (2013). *CTLA-4 and PD-1/PD-L1 Blockade: New Immunotherapeutic Modalities with Durable Clinical Benefit in Melanoma Patients*.

